# The P-glycoprotein Inhibitor GF120918 Modulates Ca^2+^-Dependent Processes and Lipid Metabolism in *Toxoplasma Gondii*

**DOI:** 10.1371/journal.pone.0010062

**Published:** 2010-04-08

**Authors:** Iveta Bottova, Ursula Sauder, Vesna Olivieri, Adrian B. Hehl, Sabrina Sonda

**Affiliations:** 1 Institute of Parasitology, University of Zurich, Zurich, Switzerland; 2 Biozentrum, University of Basel, Basel, Switzerland; Singapore Immunology Network, Singapore

## Abstract

Up-regulation of the membrane-bound efflux pump P-glycoprotein (P-gp) is associated with the phenomenon of multidrug-resistance in pathogenic organisms, including protozoan parasites. In addition, P-gp plays a role in normal physiological processes, however our understanding of these P-gp functions remains limited. In this study we investigated the effects of the P-gp inhibitor GF120918 in *Toxoplasma gondii*, a model apicomplexan parasite and an important human pathogen. We found that GF120918 treatment severely inhibited parasite invasion and replication. Further analyses of the molecular mechanisms involved revealed that the P-gp inhibitor modulated parasite motility, microneme secretion and egress from the host cell, all cellular processes known to depend on Ca^2+^ signaling in the parasite. In support of a potential role of P-gp in Ca^2+^-mediated processes, immunoelectron and fluorescence microscopy showed that *T. gondii* P-gp was localized in acidocalcisomes, the major Ca^2+^ storage in the parasite, at the plasma membrane, and in the intravacuolar tubular network. In addition, metabolic labeling of extracellular parasites revealed that inhibition or down-regulation of *T. gondii* P-gp resulted in aberrant lipid synthesis. These results suggest a crucial role of *T. gondii* P-gp in essential processes of the parasite biology and further validate the potential of P-gp activity as a target for drug development.

## Introduction

The integral membrane protein P-glycoprotein (P-gp, MDR1, ABCB1) is one of the most studied cellular transporters of the ATP-binding cassette (ABC) transporter superfamily [1]. The clinical importance of P-gp derives from the fact that over-expression of this transporter is commonly associated with the phenomenon of multidrug resistance [2], a major public health problem derived from drug-resistant cancer cells and microbial pathogens. The main function of P-gp is the export of xenobiotics from the cell, as corroborated by the findings that P-gp deficient mice are viable but show strikingly altered pharmacokinetics and increased sensitivity to a variety of drugs [3]. In addition to this well known role, an increasing amount of evidence now suggests that P-gp also participates in normal physiological processes, including the transport of steroid hormones [4] and lipid translocation (rev. in [5]).

Here we investigated the effects of the potent P-gp inhibitor GF120918 in the biology of *Toxoplasma gondii*, a model intracellular parasite and an important human pathogen, causing toxoplasmosis. Previous studies based on efflux analyses in the presence of P-gp inhibitors suggested that an active P-gp homologue is present in *T. gondii* [6], [7]. Recently, two P-gp homologues with the typical P-gp structure have been identified in the genome of the parasite (TgABC.B1 and TgABC.B2) and found to be constitutively expressed in both the vegetative and quiescent stages of *T. gondii*'s life cycle [8]. Further molecular characterization revealed that TgABC.B1 is coded by a single copy gene, expressed as a membrane-associated protein of ∼150 kDa, and constitutively present in different parasite strains [9]. Indications that *T. gondii* P-gp may be involved in key biological processes, such as replication and host cell invasion were provided by early works using P-gp inhibitors [6], [10]. However, given that these studies used host cells containing P-gp, it was not possible to discriminate between the contribution of *T. gondii* and host cell P-gp. Indeed, we recently showed that host cell P-gp plays a crucial role in *T. gondii* replication by facilitating the transport of host cholesterol to the parasite vacuole [11]. In this study we used P-gp deficient host cells [3] in parallel with pharmacological inhibition of P-gp, thereby enabling more selective insights into the specific role of *T. gondii* P-gp. Inhibition of parasite P-gp was achieved with the acridonecarboxamide derivative GF120918, a potent competitive P-gp inhibitor of the latest generation [12], [13], whose use has been widely published both *in vitro* [14] and *in vivo* [15], [16]. Importantly, GF120918 does not inhibit the P-gp-related multidrug transporters MRP1 and MRP2 [17] nor cytochrome P450 3A, a key enzyme in drug metabolism [18], and achieves adequate P-gp inhibition *in vivo* without significant side effects [13], [19].

## Results

### GF120918 inhibits parasite invasion

**Figure 1:**
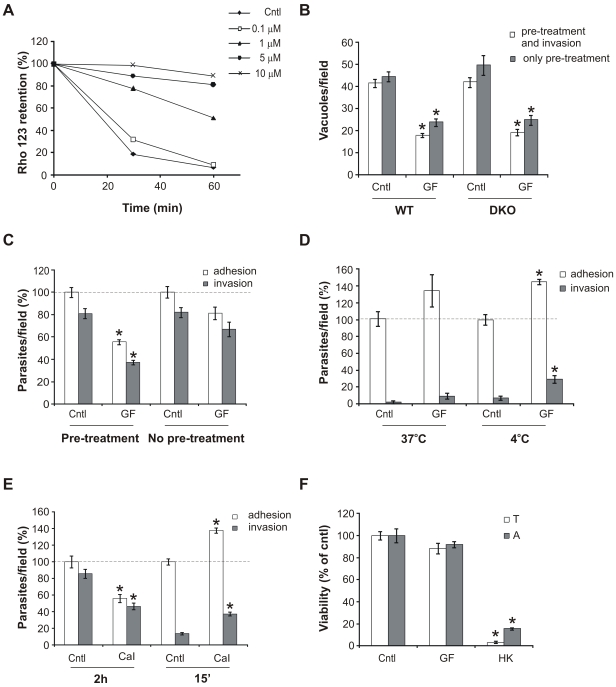
GF120918 treatment inhibits parasite invasion. A. Functionality assay of P-gp in isolated *T. gondii* treated with the indicated inhibitor concentrations as measured by time course analysis of intracellular rhodamine 123 (Rho 123) retention. Retention is expressed as percentage of mean fluorescence of intracellular Rho 123 at time 0. B. Invasion assay in presence of 10 µM GF120918 (GF) or solvent (cntl). *T. gondii* were pre-treated with GF120918 for 30 min and allowed to infect wild type (WT) or P-gp deficient (DKO) host cell monolayers in presence of GF120918 (pre-treatment and invasion, white bars). Alternatively, parasites were pre-treated with 10 µM GF120918 for 4 h, washed to remove the drug and incubated with host cells in absence of GF120918 (only pre-treatment, gray bars). Invasion was quantified by enumerating the parasite vacuoles at 24 h post infection. Data are average of vacuoles per field ± SE (n = 15) of a representative of 3 experiments. C. Adhesion/invasion assay in presence of 10 µM GF120918 (GF) or solvent (cntl). Parasites were pre-treated with GF120918 for 30 min at 37°C and allowed to infect DKO host cell monolayers for 2 h in presence of the drug (pre-treatment). Alternatively, drug pre-treatment was omitted and GF120918 added at the time of invasion (no pre-treatment). Adherent (intracellular + extracellular) and invaded (intracellular) parasites were counted after dual color immunostaining. Data are average of parasites per field ± SE (n = 15) of a representative of 3 experiments, normalized with respect to cntl adhesion and expressed as percentage. D. Adhesion/invasion assay in presence of 10 µM GF120918 (GF) or solvent (cntl). Parasites were pre-treated with GF120918 for 30 min at 37°C or 4°C and allowed to infect DKO host cell monolayers for 15 min in presence of the drug. Parasites were counted as in C. Data are average of parasites per field ± SE (n = 10) of a representative of 2 experiments, normalized with respect to cntl adhesion and expressed as percentage. E. Adhesion/invasion assay in presence of 1 µM of the Ca^2+^ ionophore A23187 (CaI) or solvent (cntl). Parasites were allowed to infect DKO host cell monolayers for 2 h or 15 min in presence of the drug. Adherent and invaded parasites were counted after dual color immunostaining. Data are average of parasites per field ± SE (n = 10) of a representative of 3 experiments, normalized with respect to cntl adhesion and expressed as percentage. F. Isolated parasites were treated with 10 µM GF120918 (GF) or solvent (cntl) for 4 h at 37°C or heat killed (HK) for 1 h at 65°C. Cell viability was assessed by measuring AlamarBlue reduction (Biosource) (A) or trypan blue exclusion (T). Results are average ± SE (*n* = 3 for A and 8 for T) and expressed as percentage of cntl viability. *, *p*<0.05.

As an obligate intracellular parasite, *T. gondii* depends completely on host cells for its survival and propagation; thus host cell invasion is an essential process in the parasite's biology. To analyze whether P-gp inhibition compromises parasite invasion, we blocked P-gp function in isolated parasites with GF120918, a potent P-gp inhibitor of the latest generation [13]. GF120918 was found to strongly hamper P-gp function in the parasite at low micromolar concentrations, as assessed by efflux analysis of the specific P-gp substrate rhodamine 123 (Fig. 1A). To analyze whether GF120918 inhibits parasite invasion, parasites were pre-treated with the inhibitor for 30 min at 37°C and allowed to infect host cells wild type (WT) or deficient in the two mouse P-gp isoforms (P-gp DKO) [3] for 4 h in presence of the drug. GF120918 was then removed and the infection was determined by counting the parasite vacuoles after 24 h incubation. GF120918 treatment reduced the number of intracellular vacuoles by 50% in both host cell types, indicating that host P-gp is not involved in parasite invasion (Fig. 1B, white bars). Importantly, the invasion inhibition was not caused by parasite lethality following compound treatment, as GF120918 did not significantly compromise parasite viability at the concentration inhibitory for invasion (Fig. 1F). To analyse whether the presence of GF120918 at the time of infection was necessary for the inhibitory effect, parasites were pre-treated with GF120918, washed and incubated with host cells in absence of the drug. Also in these experimental conditions, parasite invasion was reduced by ∼50% (Fig. 1B, grey bars), confirming that the drug inhibited parasite invasion by acting solely on the parasite. These results also showed that the invasion inhibition is not reversed by removal of the drug from the medium, suggesting that GF120918 stably inhibited the parasite target.

Having shown that GF120918 inhibited parasite invasion, we then further dissected the inhibitory effect using a sequential staining method, which allows discrimination between the processes of adhesion to a host cell and active invasion. P-gp DKO host cells were used during GF120918 treatment to exclude the contribution of any residual host P-gp activity to the observed phenotype. Parasites were pre-treated for 30 min at 37°C with GF120918 and allowed to infect host cells for 2 h in presence of the drug. In these experimental conditions parasite adhesion and invasion were reduced by ∼50% compared with control cells (Fig. 1C, pre-treatment). This dual inhibition is indicative of an effect on parasite attachment, since attachment is required for invasion [20]. Interestingly, in the absence of parasite pre-treatment, the inhibitory effect of GF120918 on parasite adhesion and invasion was less pronounced (Fig. 1C, no pre-treatment), suggesting that a time delay after adding the compound is necessary for the inhibitory effect to occur.

To further analyze the time dependency of GF120918 effect on parasite invasion, we pre-treated the parasites with the inhibitor for 30 min at 37°C or 4°C, to minimize the secretion of proteins required for invasion, and we compared adhesion and invasion after 15 min of host cell contact in the presence of the drug. Fig. 1D shows that at these short invasion times GF120918, treatment promoted both parasite adhesion and invasion compared with control cells. This time dependent GF120918 effect on parasite invasion was reminiscent of the phenotype observed upon treatment with the Ca^2+^ ionophore A23187. A23187 is a potent modulator of Ca^2+^-dependent processes in the parasite, including secretion of microneme proteins required for motility and invasion. However, prolonged stimulation of microneme secretion inhibits parasite invasion ([21], [22]), presumably due to the exhaustion of factors required for the invasion process. In agreement with these previous findings and similar to our observations in GF120918 treated cells, short incubation times with A23187 promoted parasite invasion, while prolonged A23187 exposure was detrimental (Fig. 1E).

### GF120918 treatment increases T. gondii motility

**Figure 2:**
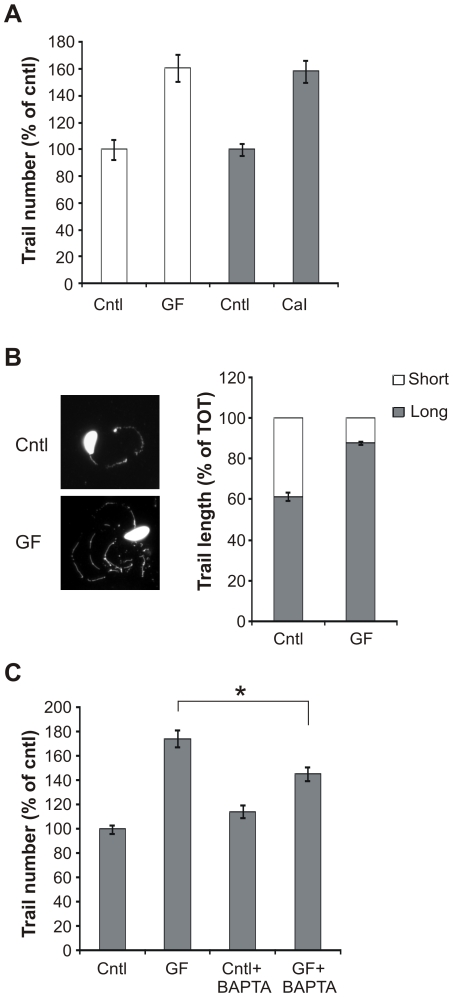
GF120918 treatment increases parasite motility. A. Parasites were treated with 10 µM GF120918 (GF, white bars), 1 µM Ca^2+^ ionophore A23187 (CaI, gray bars) or the respective solvents (cntl) and the trails deposited during gliding enumerated. Data are average of trail number per field ± SE (n = 15) of a representative of 3 experiments and expressed as percentage of cntl. B. Indirect immunofluorescence microscopy (left panel) and trail length quantification (right panel) demonstrating that the average length of trails increased with GF120918 (GF) treatment. Data are percentage of short (<10 µM) or long (>10 µM) trails per field ± SE (n = 10) of a representative of 2 experiments. C. Parasites were treated with 10 µM GF120918 (GF) in presence or absence of 100 µM BAPTA and trail deposition was quantified as before. Data are average of trail number per field ± SE (n = 10) of a representative of 2 experiments and expressed as percentage of cntl-treated parasites. *, *p*<0.05.

From the above experiments we established that GF120918 modulates parasite invasion. As GF120918 increased parasite invasion at short incubation times and the invasion process depends on parasite motility (reviewed in [23]), we next examined whether GF120918 treatment increased *T. gondii* motility as well. Parasite motility was analyzed by allowing parasites to glide on a substratum in presence or absence of GF120918 and visualizing the trails produced by parasite movement by immunofluorescence [24]. Parasites pre-treated with GF120918 for 30 min at 4°C produced more (Fig. 2A, white bars) and longer trails (Fig. 2B) compared with control cells. Parasite motility is known to be regulated by intracellular Ca^2+^ fluxes [21], [25]. To test whether the alteration of parasite motility induced by GF120918 treatment correlated with the effects of Ca^2+^ deregulation, we increased intracellular calcium levels using the Ca^2+^ ionophore A23187. In our experimental conditions, both P-gp inhibitor and A23187 incubations increased parasite motility in a comparable manner (Fig. 2A, gray bars). In addition, when parasites were co-treated with GF120918 together with the Ca^2+^ chelator BAPTA, the increase in motility was reduced compared with parasites solely treated with GF120918 (Fig. 2C). Collectively, these findings indicated that GF120918 treatment increases parasite motility and that this increase is likely to depend on alterations in parasite's Ca^2+^ fluxes.

### GF120918 treatment induces microneme secretion

**Figure 3:**
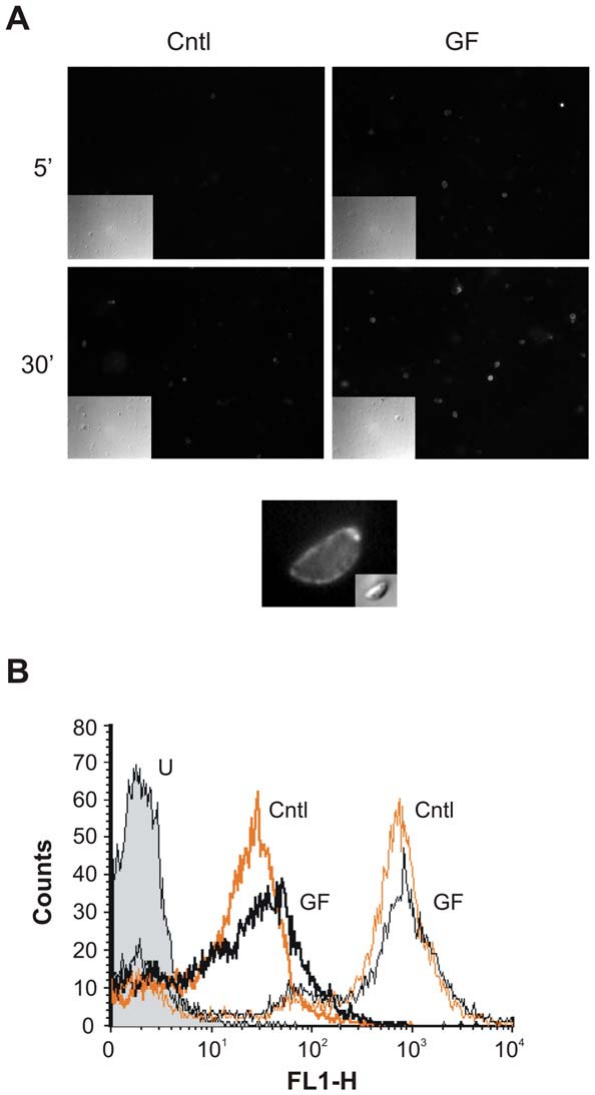
GF120918 treatment increases MIC2 secretion. A. Isolated parasites were treated with 10 µM GF120918 (GF) or solvent (cntl) for 5 or 30 min, fixed and stained with anti-MIC2 antibody without cell permeabilization. Central panel, magnification of *T. gondii* treated with GF120918 for 30 min showing MIC2 surface staining. Insets, differential interference contrast images. B. Isolated parasites treated with 10 µM GF120918 (GF) or solvent (cntl) for 30 min were stained with anti-MIC2 antibody without (thick lines) or with (thin lines) cell permeabilization and quantified by flow cytometry. Overlay histogram showing cells treated with solvent (cntl, yellow lines) or GF120918 (GF, black lines). U, unstained cells.

Secretion of micronemes, secretory organelles located at the apical end of the parasite, depends on Ca^2+^ fluxes in the parasite and it is essential for *T. gondii* motility [21]. To analyse whether GF120918 affected microneme secretion, we examined the discharge of the microneme protein MIC2, which is secreted on the parasite surface and then shed after proteolytic processing [26]. Parasites treated for 5 and 30 min with GF120918 showed a time dependent increase in MIC2 surface staining compared with control cells (Fig. 3A). Population-wide quantification by flow cytometry revealed that MIC2 surface staining in non-permeabilized cells increased following GF120918 treatment in ∼25% of the cells (Fig. 3B, thick lines). The staining was not due to cell permeabilization induced by inhibitor treatment, as detergent-permeabilized cells showed a much stronger signal (Fig. 3B, thin lines). In addition, analysis of permeabilized cells revealed that in control samples 64.44% of the total cell number (n = 10'000) showed high MIC2 signal, while GF120918 treatment decreased this number to 48.47%, suggesting a loss of cell associated MIC2 following secretion, further confirmed by Western blot analysis of MIC2 recovered in the culture supernatant (Fig. S2). Collectively, these data indicate that GF120918 treatment induced microneme secretion without increasing the permeability of parasite plasma membrane.

### GF120918 treatment induces parasite egress

**Figure 4:**
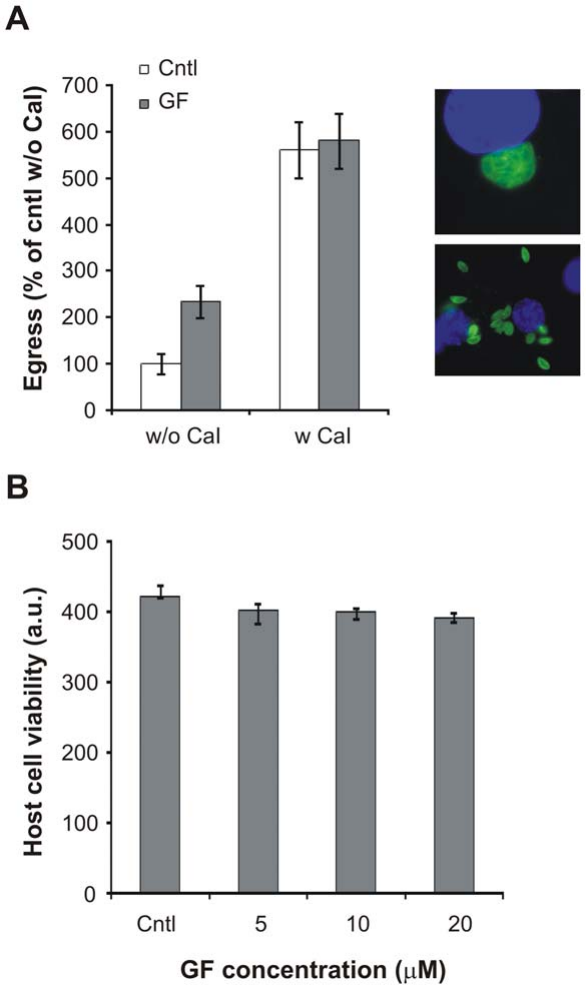
GF120918 treatment increases *T. gondii* egress. A. *T. gondii* was allowed to replicate in P-gp DKO host cells for 30 h, treated with 10 µM GF120918 (GF) or solvent (cntl) for 4 h and egress quantified after parasite immunolabeling. For Ca^2+^ ionophore (CaI)-induced egress, infected monolayers were treated with GF120918 as before and additionally incubated with 1 µM A23187 for 10 min. Data are average of egressed parasites per field ± SE (n = 20) expressed as percentage of cntl without CaI. Right panels, examples of intravacuolar and egressed parasites. B. Metabolic activity of P-gp DKO host cells treated for 24 h with solvent (cntl) or GF120918 (GF) at the indicated concentrations was assessed by measuring AlamarBlue reduction (Biosource). Results are average ± SE (*n* = 6).

As we observed that GF120918 treatment of extracellular *T. gondii* enhanced parasite motility, we reasoned that if GF120918 could activate motility also in intracellular parasites, it would promote active egress of *T. gondii* from the host cells, a process which depends on parasite motility and is triggered by changes in the host cells ionic environment [27]. When infected cultures were treated for 4 h with GF120918, a two fold increase in parasite egress was observed compared with untreated cells, whose egress rate was consistently observed at 3–10% (Fig. 4A, w/o CaI). Contrary to the natural egress which occurs at the end of the parasite lytic cycle and does not require parasite motility [28], the motility-dependent active egress is thought to be employed by the parasite as an escape mechanism from a dying host cell [29]. To determine whether the GF120918-mediated egress was caused by host cell lethality, we analyzed the viability of host cells treated with the inhibitor. Incubation with GF120918 for 24 h did not reduce host cell viability, indicating that the increased egress induced by GF120918 was not a response to inhibitor-mediated host cell toxicity (Fig. 4B).

Next we analyzed the effect of GF120918 during experimental induction of active egress. As with microneme secretion, active egress can also be induced by adding the Ca^2+^ ionophore A23187 [30], which raises intracellular calcium levels and activates parasite motility (rev. in [31]). When parasite egress was induced with A23187 incubation, a similar increase in egress was observed in presence or absence of GF120918, indicating that GF120918 treatment was not able to further increase egress when used in combination with the Ca^2+^ ionophore (Fig. 4A, w CaI).

### Intracellular localization of T. gondii P-gp

**Figure 5:**
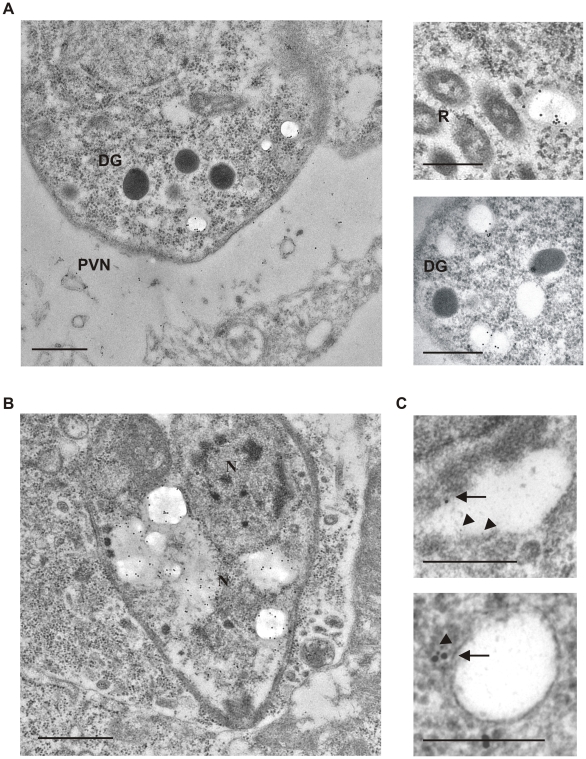
Ultrastructural localization of *T. gondii* P-gp. A. *T. gondii* was allowed to replicate in P-gp DKO host cells for 24 h and immunoelectron microscopy performed with the anti-Pgp antibody C219. PVN, parasitophorous network; DG, dense granules; R, rhoptries; N, nucleus. Scale bars, 0.5 µm. B. Examples of P-gp localization in damaged parasite vacuoles. Scale bars, 1 µm. C. Co-localization of C219 (15 nm gold, arrows) and anti-VP1 (5 nm gold, arrowheads). Scale bars, 0.5 µm.

We showed that treatment of *T. gondii* with the P-gp inhibitor GF120918 strongly correlated with alterations in Ca^2+^-dependent processes; thus, it is possible that P-gp plays a role in Ca^2+^ regulation in this parasite. To investigate this hypothesis, we analyzed whether P-gp localized in parasite organelles involved in Ca^2+^ homeostasis. For this aim we used the P-gp-specific monoclonal antibody C219, which recognizes a conserved epitope also present in the *T. gondii* orthologue (Genbank accession no. AAZ04382) (Fig. S1B). Western blot analysis confirmed that the antibody reacts with a parasite protein corresponding to the predicted mass of P-gp (Fig. S1A). Immunoelectron microscopy of infected cells showed a distinct staining concentrated in conspicuous membrane-bound electron-lucent vesicles of 100–400 nm diameter. The plasma membrane of the parasite was less intensely labeled with this antibody, and no staining of other vesicular structures, such as rhoptries and dense granules was observed (Fig. 5A). In addition, P-gp labeling was found in the tubulovesicular network within the parasite vacuole. Strikingly, P-gp staining drastically increased in the rare vacuoles which contained dying parasites, characterized by high intracellular vacuolization, plasma membrane rupture and release of cytosolic content in the parasite vacuolar space (Fig. 5B).

The P-gp labeled electron-lucent vesicles morphologically resembled acidocalcisomes, the largest intracellular store of Ca^2+^ in the parasite [32].Co-immunostaining with antibodies against the acidocalcisome marker VP1 [33] revealed that both proteins are indeed present in the same structures (Fig. 5C).

**Figure 6:**
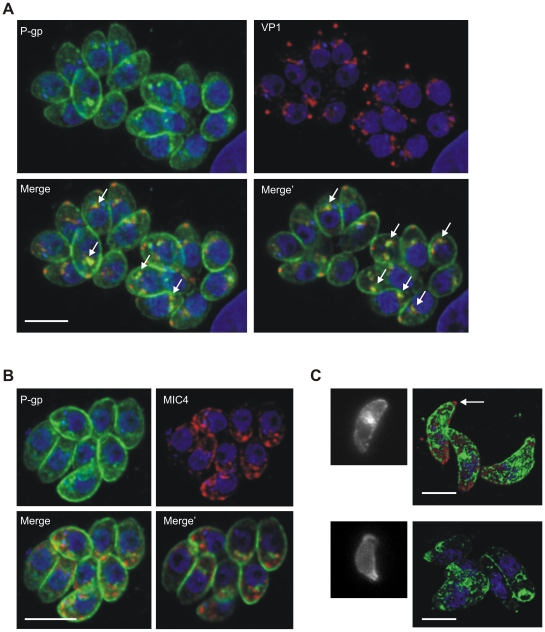
Intracellular localization of *T. gondii* P-gp. Confocal microscopy of intracellular *T. gondii* at 24 h post infection. A. Dual staining using polyclonal mouse anti-P-gp minigene (green) and anti-VP1 (red) antibodies showing partial co-localization of the two proteins (arrows). Nuclear DNA was stained with DAPI. Merge, maximum projection; Merge', single optical section of the deconvolved image stacks. B. Dual staining using anti-P-gp minigene (green) and anti-MIC4 (red) antibodies. Merge, maximum projection; Merge', single optical section of the deconvolved image stacks. C. Wide field fluorescence (left panels) and 3D reconstructed confocal (right panels) micrographs showing differential P-gp distribution in extracellular parasites stained with anti-P-gp minigene antibody (green). The arrow indicates the absence of P-gp staining in the conoid area labeled with anti-*T. gondii* antibody (red). Nuclear DNA was stained with DAPI (blue). Scale bars: 5 µm.

Next, we investigated the localization of P-gp by immunofluorescence analysis. As the monoclonal antibody C219 is suitable for this technique only in case of P-gp overexpression, we engineered a P-gp minigene composed of three sequences of the parasite protein showing low degree of similarity with the mammalian P-gp (Fig S1B), and which encompassed the peptide sequence used in a previous study to raise anti-*T. gondii* P-gp antibodies [9]. Similar to the C219 antibody, the immune serum against P-gp minigene reacted with a band of mass expected for P-gp (Fig. S1A). As previously reported [9], lower molecular weight bands were also detected; it is currently not known if they constitute protein degradation or processing products. Immunofluorescence analysis of *T. gondii* infected cells using the P-gp minigene antibody showed a strong labeling of distinct intracellular structures distributed throughout the cell and of plasma membrane (Fig. 6A). Similar to the observations with electron microscopy, these intracellular structures partially co-localized with VP1 (Fig. 6A) but not with the microneme protein MIC4 (Fig. 6B), strongly suggesting that P-gp is indeed localized in acidocalcisomes.

Interestingly, when extracellular *T. gondii* were probed with the P-gp minigene antibody, a subgroup of parasites showed P-gp labeling exclusively on the plasma membrane and labeling of the internal structures was no longer evident. In addition, the staining concentrated at the apical end of the parasite and formed a collar around the conoid, which in turn remained unlabeled (Fig. 6C). These results suggest that P-gp transiently re-localized in extracellular parasites.

### GF120918 treatment inhibits T. gondii replication and lipid synthesis

**Figure 7:**
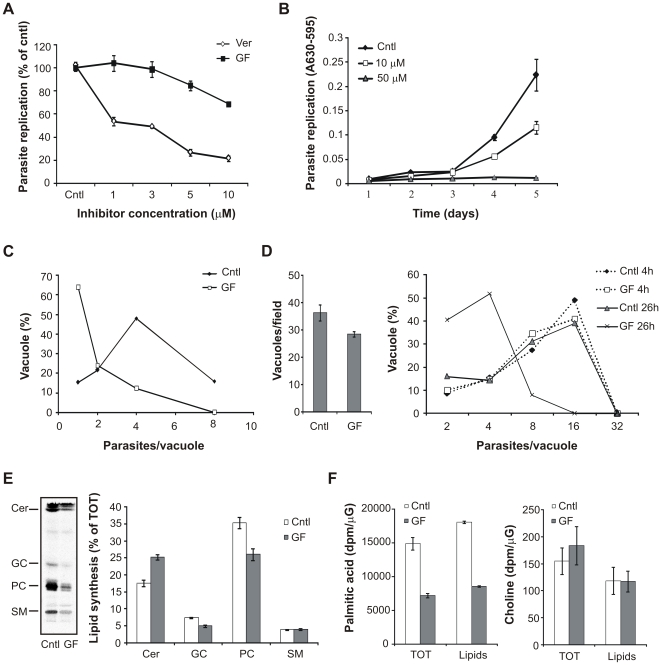
GF120918 treatment inhibits parasite replication and lipid synthesis. A. *T. gondii* were allowed to infect P-gp DKO host cells and subsequently treated with the indicated concentrations of GF120918 (GF) and verapamil (Ver). Parasite burden was quantified 48 h post infection by a colorimetric assay measuring the amount of parasite-expressed β-galactosidase. Results are expressed as percentage of parasite number in control untreated cells ± SE (n = 5). B. Time course of parasite replication inhibition by verapamil. *T. gondii* infected host cells were treated with the indicated concentrations of verapamil or solvent (cntl). Parasite burden was quantified at the indicated times post infection as described above. Results are expressed as percentage of parasite number in control untreated cells ± SE (n = 3). C. *T. gondii* were treated with 10 µM GF120918 (GF) or solvent (cntl) for 4 h, washed and incubated with P-gp DKO host cells for 24 h in absence of the drug. Intracellular parasites were quantified by direct counting after immunostaining. The distribution of the parasite number in single vacuoles is expressed as percentage of total vacuoles examined (n>100). D. Left panel: parasites were allowed to infect host cell monolayers for 4 h, followed by 10 µM GF120918 (GF) or solvent (cntl) treatment for additional 26 h. Vacuoles containing at least 2 parasites were enumerated at 30 h post infection after immunostaining. Data are average of vacuoles per field ± SE (n = 10). Right panel: parasites were allowed to infect host cell monolayers for 4 h, and treated with 10 µM GF120918 (GF) or solvent (cntl) for 26 h or for the last 4 of the 30 h incubation. Intracellular parasites were quantified by direct counting after immunostaining. The distribution of the parasite number in single vacuoles is expressed as percentage of total vacuoles examined (n>100). E. Isolated parasites were labeled with 0.5 µCi/mL of [^3^H]palmitic acid for 3 h in presence of 10 µM GF120918 (GF) or solvent (cntl) and lipid were extracted. Left panel, TLC separation of de novo synthesized lipids. Lipid standard used: ceramide (Cer), glucosylceramide (GC), phosphatidylcholine (PC), sphingomyelin (SM). Right panel, quantification of the relative amount of newly synthesized lipid species expressed as percentage of total lipids. Data are average ± SE (n = 3). F. Isolated parasites were labeled with 0.5 µCi/mL of [^3^H]palmitic acid or 4 µCi/mL [^3^H]choline for 3 h in presence of 10 µM GF120918 (GF) or solvent (cntl). [^3^H] incorporation was measured in both whole cell (TOT) and lipid extracts by liquid scintillation and normalized by protein content. Data are average ± SE (n = 6).

To determine whether GF120918 treatment affected parasite replication, P-gp DKO host cells were infected with *T. gondii* and subsequently treated with different concentrations of the inhibitor. Analysis of parasite burden 48 h post infection revealed a marked dose-dependent inhibition of parasite replication (Fig. 7A). Immunofluorescence analysis did not show any cells positive for the bradyzoite-specific antigen BAG1 (data not shown), indicating that GF120918 treatment reduced parasite replication without triggering parasite stage conversion to the quiescent bradyzoite form. Treatment with verapamil, an earlier generation P-gp inhibitor reported to be less potent than GF120918 [34], was less effective in blocking parasite replication. However, longer incubation times with verapamil strongly inhibited *T. gondii* replication as well (Fig. 7B), without affecting host cell viability (data not shown). In addition, GF120918-mediated inhibition of parasite replication was not reversed following removal of the drug: parasites treated with the inhibitor prior to contact with host cells not only showed defective invasion (Fig. 1B), but also formed smaller vacuoles containing fewer parasites than control cells after 24 h incubation in the absence of the drug (Fig. 7C).

We previously showed that GF120918 induced egress of intracellular parasites, albeit less potently than the Ca^2+^ ionophore A23187 (Fig. 4A). Next we determined whether increased parasite egress contributed to the reduced parasite burden in cell cultures. Freshly infected host cells were treated with GF120918 and parasite vacuoles were enumerated after 30 h incubation. Inhibitor treatment slightly decreased the number of vacuoles in infected cells (Fig. 7D, left panel), possibly due to egressed parasites and/or inhibition of host cell re-invasion (Fig. 1B, C). However, analysis of parasite burden revealed a much more pronounced decrease in the number of parasites contained in the vacuoles (Fig. 7D, right panel, solid lines). In addition, when infected cells were treated with GF120918 only in the last 4 of the 30 h incubation, no difference in parasite distribution was observed (Fig. 7D, right panel, dashed lines), indicating that GF120918 does not selectively induce the egress of vacuoles containing high numbers of parasites.

Collectively, these results suggest that GF120918-induced egress may contribute to, but is not sufficient to account for the reduced parasite burden observed in these experiments and that inhibitory mechanisms directly acting on parasite replication are likely to occur.

In search for the molecular mechanisms responsible for the GF120918-mediated inhibition of parasite replication, we analyzed the effect of the inhibitor on *T. gondii* lipid metabolism, a critical process for proper parasite replication. As we recently showed that host P-gp is involved in cholesterol transport from the host cell to the parasite vacuoles [11], we hypothesized that the defective parasite replication observed during *T. gondii* treatment with the P-gp inhibitor may derive from impaired lipid transport and/or synthesis. To test this, we treated isolated parasites with the inhibitor and monitored lipid synthesis after metabolic labeling. The fatty acid precursor [^3^H]palmitic acid was readily incorporated in phospho- and sphingolipid species of control parasites (Fig. 7E). However, incubation with GF120918 induced a general decrease in parasite lipid synthesis. Interestingly, the inhibitor did not reduce the synthesis of all labeled lipids in a similar manner, but differently affected the relative amount of the lipid species, with PC synthesis being the most down-regulated while ceramide synthesis was increased compared with control cells. Next, we determined whether GF120918 affected not only lipid synthesis but also lipid precursor uptake. Liquid scintillation analysis revealed that inhibitor treatment strongly inhibited the incorporation of [^3^H]palmitic acid in both total cell extracts and lipid fraction (Fig. 7F). This reduced incorporation was specific for [^3^H]palmitic acid as incorporation of [^3^H]choline was not affected by GF120918 treatment. Collectively, these data show that GF120918-induced inhibition of *T. gondii* replication was likely mediated by a reduction in parasite lipid uptake and synthesis.

### Down-regulation of T. gondii P-gp correlates with reduced parasite replication and lipid synthesis

**Figure 8:**
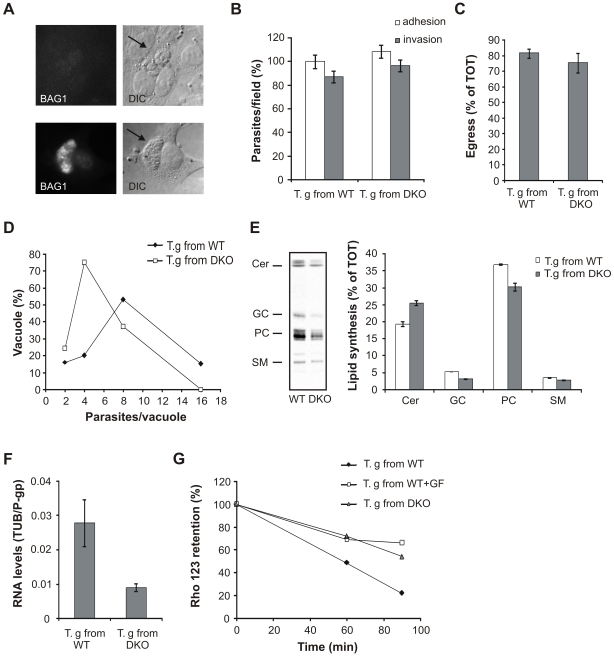
Down-regulation of *T. gondii* P-gp correlates with decreased lipid synthesis. A. Immunoflorescence analysis using antibodies against the bradyzoite-specific antigen BAG1 showing the absence of parasite stage conversion into bradyzoites after more than 4 lysis cycles in P-gp DKO host cells (upper panels). Lower panels, positive control of parasites induced to differentiate by alkaline treatment as described in the Materials and Methods section. Arrows, parasite vacuoles. DIC, differential interference contrast images. B. Adhesion/invasion assay in WT host cells of *T. gondii* isolated from P-gp WT or DKO host cells after more than 4 lysis cycles. Adherent and invaded parasites were counted after dual color immunostaining. Data are average of parasites per field ± SE (n = 10) and expressed as percentage of cntl adhesion of *T. gondii* maintained in WT host cells. C. The same isolated parasites were allowed to replicate in WT host cells for 30 h, following induction of egress with 1 µM A23187 for 10 min. Data are average of egressed parasites per field ± SE (n = 15) expressed as percentage of the total (egressed + intravacuolar) parasites. D. Parasites isolated from P-gp WT or DKO host cells after more than 4 lysis cycles were incubated with WT host cells for 24 h. Intracellular parasites were quantified by direct counting after immunostaining. The distribution of the parasite number in single vacuoles is expressed as percentage of total vacuoles examined (n>100). E. Parasites isolated from P-gp WT or DKO host cells after more than 4 lysis cycles were labeled with 0.5 µCi/mL of [^3^H]palmitic acid for 3 h and lipid were extracted. Left panel, TLC separation of de novo synthesized lipids. Lipid standard used: ceramide (Cer), glucosylceramide (GC), phosphatidylcholine (PC), sphingomyelin (SM). Right panel, quantification of the relative amount of newly synthesized lipid species expressed as percentage of total lipids. Data are average ± SE (n = 3). F. Semi-quantitative real time PCR of P-gp expression levels in parasites isolated from P-gp WT or DKO host cells after more than 4 lysis cycles. Gene expression levels were given as values in arbitrary units relative to the amount of the constitutively expressed house-keeping gene tubulin. Data are average ± SE (n = 5). G. Functionality assay of P-gp in *T. gondii* isolated from P-gp WT or DKO host cells after more than 4 lysis cycles. Parasites from WT host cells were treated with 5 µM GF120918 (GF) as positive control for inhibition of P-gp functionality. Intracellular rhodamine 123 (Rho 123) retention is expressed as percentage of mean fluorescence of intracellular Rho 123 at time 0.

While *T. gondii* is able to grow in P-gp DKO host cells, its replication rate is reduced due to defective cholesterol trafficking to the parasite vacuole [11]. When we analyzed *T. gondii* maintained for more than 4 lysis cycles in P-gp DKO host cells, we found that the sub-optimal growth conditions did not trigger parasite stage conversion to the quiescent bradyzoite form (Fig. 8A). In addition, parasites isolated from P-gp DKO host cells after more than 4 passages were able to infect (Fig. 8B) and to egress from (Fig. 8C) new host cells as efficiently as parasites maintained in WT host cells. These finding suggest that the lower parasite burden observed was not caused by compromised infection or exit from host cells, but by a specific defect in replication, consistent with the limited access to host cholesterol demonstrated previously [11]. However, when parasites isolated from P-gp DKO host cells were allowed to infect WT host cells with a normal cholesterol trafficking, their replication rate remained slower compared with parasites isolated from WT host cells, as monitored up to 72 h post infection (Fig. 8D and not shown). While it is conceivable that cholesterol storage still depleted in the parasite after prolonged growth in P-gp DKO host cells affected *T. gondii* replication, it is also possible that lipid transport and/or synthesis are altered in these parasites. To test this hypothesis, we monitored the lipid synthesis in *T. gondii* isolated from P-gp DKO host cells after more than 4 lysis cycles. Surprisingly, [^3^H]palmitic acid labeled cells showed an overall decrease in lipid synthesis with a profile similar to what was observed during P-gp inhibition with GF120918 (Fig. 8E). To investigate whether the inhibition of lipid synthesis correlated with inhibition of *T. gondii* P-gp, we performed real-time PCR (Fig. 8F) and P-gp functionality assay (Fig. 8G) on parasites isolated from WT or P-gp DKO host cells. Both approaches indicated that P-gp expression and activity were reduced in parasites grown in P-gp DKO host cells, supporting the correlation between lipid synthesis and P-gp activity revealed by pharmacological P-gp inhibition.

## Discussion

P-gp has been shown to be a major mechanism of multidrug resistance in pathogenic organisms, including protozoan parasites (rev. in [35]). However, the physiological roles of P-gp in the absence of drug pressure remain poorly understood. In the present study we investigated the effects of the potent, third generation P-gp inhibitor GF120918 in the pathogenic parasite *T. gondii*. We found a dual role of GF120918 in the parasite physiology, namely in Ca^2+^-dependent processes and lipid synthesis.

### GF120918 treatment and Ca^2+^-regulated processes

Ca^2+^ fluxes have been shown to play a key role in *T. gondii* invasion by regulating the secretion of microneme proteins, which serve as a bridge between the internal actin-myosin motor system and external substrates, and thus mediate both *T. gondii* adhesion and movement [36]. However, the molecular mechanisms regulating Ca^2+^ homeostasis in the parasite are not completely elucidated [37]. Here we showed that GF120918 treatment induced the secretion of the microneme protein MIC2 in absence of contact with host cells. In addition, increased MIC2 secretion correlated with increased parasite motility, in terms of number and length of trails left by the parasite while gliding on a substrate. Increased motility was not reported in a previous study using an early generation P-gp inhibitor [6]. While the different methods used by the authors to quantify parasite movement could account for this discrepancy, another explanation derives from different potency and binding capacity of the inhibitors to P-gp, which is also suggested by the different reversibility of the compounds observed in replication and invasion assays. Consistent with a role in promoting Ca^2+^-mediated motility, GF120918 treatment induced *T. gondii* egress from infected host cells, a process known to depend on parasite motility and Ca^2+^ fluxes [27].

Increased *T. gondii* motility has been reported only rarely, and is generally characterized by fast and unregulated movement not resulting in productive locomotion. Examples are treatments with jasplakinolide, an actin filament stabilizer [38], or selected small molecules identified in a screen for parasite invasion inhibitors [39]. However treatment with calmidazolium, which increased intracellular Ca^2+^ levels in the parasite, induced both microneme secretion and productive motility [40]. The mechanism of calmidazolium-mediated increase of Ca^2+^ levels has not been identified in the parasite as yet; however the fact that chelation of extracellular Ca^2+^ decreased the calmidazolium-induced parasite motility led to the hypothesis that the compound may promote the influx of extracellular Ca^2+^ by modulating a Ca^2+^ transporter localized on the parasite plasma membrane [40]. Importantly, in our experimental conditions, extracellular Ca^2+^chelation decreased the GF120918-induced parasite motility as well. The striking similarity of the calmidazolium and GF120918 effect on parasite motility strongly suggests that the two compounds operate on a similar molecular mechanism and reveal a possible involvement of P-gp in Ca^2+^ signaling in the parasite. In addition, it is possible that calmidazolium, which is marketed as a calmodulin inhibitor, induced the *T. gondii* phenotype by interfering with P-gp function, as Pgp is inactivated by calmodulin antagonists [41]. Conversely, GF120918 has not been reported thus far to interfere with Ca^2+^ transport or Ca^2+^-dependent processes. Analysis of P-gp-dependent Ca^2+^ fluxes is limited by the fact that commonly used Ca^2+^ indicators are known P-gp substrates and are actively exported from the cell interior [42]. However studies performed with radiolabeled Ca^2+^ in mammalian cells indicated that this ion accumulated more in P-gp over-expressing than in control cells [43]–[45]. Two P-type Ca^2+^ transporters, the plasma membrane Ca^2+^-ATPase (PMCA) and the sarcoplasmic reticulum Ca^2+^-ATPase (SERCA), play a crucial role in maintaining Ca^2+^ homeostasis. Both proteins counter-transport protons and alteration of intracellular pH has been shown to produce changes in Ca^2+^ levels (Rev. in [46]). Interestingly, P-gp expression has been associated with outward proton movement and intracellular alkalinization as a consequence of increased Cl^−^/H^+^ antiport [47]; similar data were also obtained in the analysis of human P-gp over-expression in yeast cells [48] and in the characterization of a bacterial P-gp homologue [49]. While the direct mechanism regulating the P-gp-mediated ion transport is not known, it is conceivable that P-gp may play a role in Ca^2+^ homeostasis by regulating cellular concentration of protons and pH-dependent Ca^2+^ transporters. Ca^2+^ regulation via PMCA and SERCA homologues is operational in *T. gondii* (rev. in [37], [50]). Importantly, a PMCA-type Ca^2+^-ATPase (TgA1) involved in Ca^2+^ influx, is localized to acidocalcisomes, the largest Ca^2+^ storage in *T. gondii* (rev. in [32]). TgA1 deficiency reduced *T. gondii* invasion and decreased parasite virulence [33], confirming that these organelles play an essential role in Ca^2+^ homeostasis and cellular processes in the parasite. Surprisingly, our localization data revealed that P-gp was found not only on the plasma membrane of the parasite, but also in organelles morphologically resembling acidocalcisomes and labeled with the acidocalcisome marker VP1, further supporting a P-gp involvement in Ca^2+^ regulation. Similarly to the observations with the acidocalcisome marker type I vacuolar-type H^+^-pyrophosphatase [51], P-gp transiently re-localized in a collar-like structure at the apical end of extracellular parasites. Thus, we can propose a model where P-gp may participate in the Ca^2+^ homeostasis via facilitating Ca^2+^ uptake in acidocalcisomes by contributing to the establishment of the proton gradient in the organelles and, in addition, via promoting the function of TgA1. In addition, P-gp may also facilitate the activity of PMCA and its inhibition would promote the entry of extracellular Ca^2+^ and increase parasite motility. In support of this hypothesis, chelation of extracellular Ca^2+^ reduced GF120918-stimulated parasite motility.

P-gp has also been proposed as a regulator for Cl^−^ selective channels with a role in cell volume regulation [52]. As acidocalcisomes have been implicated in maintaining ionic homeostasis and regulating cell volume in Leishmania parasites [53], it is tempting to speculate that *T. gondii* P-gp present in these organelles may contribute to this regulatory function as well.

One intriguing finding of *T. gondii* treatment with GF120918 was the inhibition of host cell invasion despite increased microneme secretion. Microneme secretion is necessary for a successful host cell invasion; however several results suggest that it is not sufficient to promote this process. Indeed, strong stimulation of micronemes via prolonged Ca^2+^ increase inhibits parasite invasion ([21], [22] and our study), presumably due to the exhaustion of required invasion factors. A similar effect on *T. gondii* invasion we monitored following GF120918 and Ca^2+^ ionophore incubation suggested that a similar mechanism is responsible for the observed time dependent inhibition. In addition, *T. gondii* deficient for the acidocalcisome Ca^2+^ transporter TgA1 also showed increased and disregulated microneme secretion and invasion inhibition [33]. Interestingly, no invasion defect was observed in *T. gondii* with down-regulated P-gp expression, suggesting either that the reduced P-gp activity may still be sufficient to guarantee a normal infection rate, or that compensatory mechanisms are in place to maintain proper Ca^2+^ regulation.

### GF120918 treatment and parasite replication

Our previous work demonstrated that functional host cell P-gp is required for normal parasite replication [11]. In contrast with mammalian cells where P-gp inhibition or absence does not affect cell division, in this study we showed that GF120918 treatment inhibits parasite replication. Our analyses also indicated that the replication inhibition was likely caused by defective lipid synthesis, as GF120918 treatment selectively reduced palmitic acid incorporation in both total cell and lipid extracts. Conversely the normal choline incorporation in presence of GF120918 treatment suggested that the observed defect in palmitic acid incorporation is likely to occur also at the level of fatty acid uptake. The role of P-gp as a lipid transporter/flippase has been confirmed using labeled short chain lipid analogues [5]. In addition, the recently resolved X-ray structure of P-gp revealed the presence of portals open to both the cytoplasm and the inner leaflet of the plasma membrane, consistent with a function as a lipid flippase [54]. However, experiments with naturally occurring lipids are scarce and the physiological role of P-gp in endogenous lipid transport is still uncharacterized, with the only exception of platelet-activating factor (PAF) [55]. *T. gondii* has been shown to scavenge not only cholesterol [56], but also a variety of lipids from the host cells [57]. While the mechanisms by which host–derived lipids reach the parasite are not completely elucidated, the formation of H.O.S.T., a unique system of tubular structures, has been proposed to be a key event to sequester endo-lysosomes from the host cytoplasm into the parasite vacuole, thus providing *T. gondii* with cholesterol and possibly other lipids [58]. As our analyses showed that P-gp localized also in the tubular structures forming the intravacuolar network, it is tempting to speculate that parasite P-gp is involved in the uptake of host–derived lipids, either directly or via modulation of other transporters. A broad analysis of lipid transport by *T. gondii* P-gp and related transporters will be an important area for future investigation.

Quite surprisingly, our localization studies also revealed an increased P-gp labeling in damaged parasite vacuoles, thus raising the question of a possible role of P-gp during cellular stress in the parasite. In support of this hypothesis, increased P-gp expression was observed in mammalian cells after radiation exposure and was associated with resistance to apoptotic signals and radioprotection [59], indicating that P-gp plays a role not only in cell protection during xenobiotic exposure, but also in cell survival during cell damage.

Finally, we found unexpectedly that parasites grown in P-gp deficient host cells had reduced P-gp expression. The molecular mechanisms leading to P-gp down-regulation remain to be determined. One possibility is that the reduced cholesterol content found in these parasites [11] affected P-gp expression, as cholesterol depletion has been shown to down-regulate P-gp expression [60]. Alternatively, the slower *T. gondii* replication rate observed during infection of P-gp deficient host cells may result in reduced P-gp expression. Consistent with this hypothesis, less virulent *T. gondii* strains which are characterized by a slower replication rate showed a reduced P-gp expression compared with the virulent and fast replicating RH strain [9]. Finally, similar to the observations in GF120918 treated cells, *T. gondii* with down-regulated P-gp expression showed reduced lipid synthesis. This correlation strongly supports a role of P-gp in lipid metabolism; however, a contribution of slower parasite metabolism to the observed compromised synthetic activity cannot be excluded.

In summary, this study demonstrated that GF120918 treatment potently inhibits parasite invasion and replication, thus validating the potential of P-gp inhibitors as a therapeutic strategy against *T. gondii*. In addition, we also showed that GF120918 affected Ca^2+^-dependent processes and lipid metabolism in the parasite. While our data on P-gp localization and expression are in support for a direct involvement of P-gp in these events, additional investigations using genetic approaches will be required to exclude any contribution of possible GF120918-mediated off-target effects and to further our understanding of the function of P-gp and possibly other ABC transporters in the biology of *T. gondii*.

## Materials and Methods

### Biochemical reagents

Unless otherwise stated, all chemicals were purchased from Sigma and cell culture reagents from Gibco-BRL. GF120918 was a kind gift of GlaxoSmithKline. Reagents stock solutions were prepared at the following concentrations: 2 mM GF120918 in DMSO, 10 mM BAPTA (1,2-bis (2-aminophenoxy) ethane-N, N, N', N' tetracetic acid) in 0.3 M NaHCO_3_, 1 mM A23187 in DMSO, 1 mg/mL rhodamine 123 in EtOH, 4.1 mM verapamil in PBS. Reagents were freshly diluted to the concentrations required for the individual experiment. Radiolabeled palmitic acid and choline were purchased from Amersham Pharmacia Biotech. Anti-P-gp monoclonal antibody C219 was purchased from Alexis Biochemicals; anti-*T. gondii* MIC2 and VP1 were a kind gift from J.F. Dubremetz (Université de Montpellier 2, Montpellier, France) and S. Moreno (University of Georgia, Athens, GA), respectively. Conjugated secondary antibodies were from Invitrogen. Lipid standards were from Avanti Polar Lipids, Alabaster, Alabama.

### Mammalian cell and parasite culture

Mouse embryonic fibroblasts (MEF) double knocked out (DKO) for P-gp, (77.1, Mdr1a^-/-^/Mdr1b^-/-^) [3] and parental cells were kindly provided by A. Schinkel (The Netherlands Cancer Institute, Amsterdam, The Nederlands). Cells were routinely cultured in Dulbecco's modified Eagle medium (DMEM) supplemented with 10% fetal calf serum (FCS), 2 mM glutamine, 50 U of penicillin/mL, and 50 µg of streptomycin/mL at 37°C with 5% CO_2_. β-galactosidase expressing *T. gondii* were a kind gift from J. Boothroyd (Stanford University School of Medicine, Stanford, CA). *T*. *gondii* tachyzoites of the RH strain expressing *Escherichia coli* β-galactosidase [61] were used in this study. Parasites were maintained by serial passages in MEF, harvested from infected host cells by passage through a 26-gauge needle and purified by separation on Sephadex-G25 columns (Amersham) as described [62]. Purified parasites were counted in a hemocytometer chamber and used for a new cycle of host cell invasion.

For determination of parasite invasion, parasite were pre-treated with 10 µM GF120918 for 30 min at 37°C and allowed to infect for 4 h host cell monolayers at a multiplicity of infection (MOI) of 3 in presence of the drug. Alternatively, parasites were pre-treated with 10 µM GF120918 for 4 h, washed, and allowed to infect host cell monolayers in absence of the drug. After removal of residual extracellular parasites, infected cells were incubated for 20 h in absence of the drug and invasion was quantified by enumerating the parasite vacuoles per field.

For adhesion/invasion assay, parasites were pre-treated with 10 µM GF120918 for 30 min at 37°C or 4°C, incubated with host cell monolayers at MOI 3 for 15 min at room temperature to settle on the host cells and allowed to infect for 2 h or 15 min in a 37°C incubator in presence of the drug. Alternatively, drug pre-treatment was omitted and 10 µM GF120918 added at the time of invasion for 2 h incubation. Parasites were analyzed by dual color immunostaining with anti-*T. gondii* antiserum to differentiate adherent (extracellular + intracellular) and invaded (intracellular) parasites, as described [63]. A minimum of 20 parasites were present per field examined.

For determination of parasite replication, host cell monolayers were infected at a MOI of 3; parasite burden was quantified after 24 hrs by direct parasite counting or after 48 or 72 hrs by colorimetric detection of parasite β-galactosidase using chlorophenol red-β-D galactopyranoside as substrate, as described [64]. During inhibitor analyses, GF120918 was added 2 h after infection at the concentration indicated in the figure legends.

Bradyzoite differentiation was induced in vitro by alkaline treatment as described [65]. Briefly, *T. gondii* tachyzoites were allowed to infect host cell monolayer for 2 h and then the medium was replaced with RPMI-1640 containing 1 g/L NaHCO_3_ and 50 mM tricine that had been adjusted to pH 8.1 with NaOH. Cultures were incubated at 37°C and ambient CO_2_ for 4 days.

### Cell viability assay

The metabolic activity of *T. gondii* and mammalian cells was quantified using the AlamarBlue® assay (Biosource, Camarillo, CA). Briefly, 1×10^7^ parasites were incubated at 37°C for 4 h with 10 µM GF120918 or heat killed at 65°C for 1 h and processed according to the manufacturer instructions. Mammalian cells were grown to confluence in 96 well plates, incubated for 24 h with GF120918 at the concentrations indicated in the figure legend, and processed according to the manufacturer instructions. Viability of parasites was also tested by trypan blue exclusion.

### P-glycoprotein functional assay

P-gp activity was assessed by cellular retention of the P-gp substrate rhodamine 123 (Rho). Briefly, cells were incubated with 0.5 mg/mL Rho in PBS for 30 min at 37°C in presence or absence of GF120918, at the concentrations indicated in the figure legends. After washing in PBS, cells were incubated in medium at 37°C in absence or presence of the inhibitor for the time points indicated in the figure legend. The kinetic of rhodamine retention was quantified using a FACSCalibur flow cytometer (Becton & Dickinson, Basel, Switzerland).

### Motility assay

Motility was assayed by trail deposition visualization, as described [39] with the following modifications. Freshly harvested parasites were resuspended in HBSS containing 1% FCS and 10 mM Hepes, pre-treated with 10 µM GF120918 or DMSO for 15 min at 4°C, then added to poly-lysine coated slides (Thermo Fisher, Portsmouth, NH) for 15 min at room temperature and allowed to glide for 30 min at 37°C. After buffer removal, parasite trails were fixed in PBS containing 3.6% formaldehyde, blocked and stained with anti-*T. gondii* antiserum, 1∶400 dilution, followed by Alexa 488-conjugated goat anti-rabbit IgG, 1∶200 dilution.

### Egress

Egress was assayed as described [66], with the following modifications. P-gp DKO MEF monolayers were grown on 10-well glass slides (Thermo Fisher, Portsmouth, NH), infected with *T. gondii* at an MOI of 3 and parasites were allowed to replicate for 30 h at 37°C. 10 µM GF120918 was added in the last 4 h of replication, then cells were fixed with 3.6% formaldehyde, blocked and stained with anti-*T. gondii* antiserum, 1∶2000 dilution, followed by Alexa 488-conjugated goat anti-rabbit IgG,1∶200 dilution. For induced egress, infected monolayers were treated with 10 µM GF120918 as before, washed, incubated with 1 µM A23187 in warm HBSS buffer for 10 min at 37°C and stained as described before. Lysed and intact vacuoles were quantified and egress was expressed as percentage of lysed vacuoles out of the total vacuoles present in a given field.

### Antibody production

*T. gondii* P-gp minigene was obtained by fusing three sequences of the parasite protein (aa 1–52, 295–331, 625–755) showing low degree of similarity with the mammalian P-gp. Oligonucleotides (5′-3′ orientation) used in this study were:

s1 BamHI cgggatccATGGCCACCTCAGACGACTCATC, as1 XbaI gcTCTAGA TTCT GTTCCAGAGACGAAGTGGAAC, s2 XbaI gctctagaGGACAAGTGATCTCCGACGGA

TTG, as2 EcoRI CGGAATTC AGCGTCGCCACCCTGAAACTCCAG, s3 EcoRI CGGAATTCATGAAAGACGAGAGCGGTCTG, as3 HindIII CCCAAGCTTAGGCCAGTAG

CGGAGAGCGAG.

The P-gp minigene was inserted downstream of the maltose binding protein (MBP) in the plasmid vector pMal-2Cx (New England Biolabs, MA). The fusion protein was produced in transgenic *Escherichia coli* by induction with 0.3 mM IPTG (isopropyl-β-D-thiogalactopyranoside) for 2 h at 37°C. Following protein isolation by affinity purification on amylose resin according to the manufacturers protocol (New England Biolabs, MA), P-gp minigene was dialyzed, lyophilized and 150 µg used to immunize intraperitoneally with RIBI adjuvant (Corixa, Hamilton MT, USA) NMRI mice on days 0, 15, and 30 and 50.

### Immunofluorescence analysis

Host cells infected with *T. gondii* or extracellular parasites were fixed in 3.6% formaldehyde, permeabilized with 0.2% Triton X-100 in PBS for 20 min, when indicated in the figure legends, blocked and incubated with primary antibodies for 1 h. The primary antibodies used in this study were: anti-*T. gondii* tachyzoite rabbit antiserum [67], 1∶2000 dilution, anti-*T. gondii* MIC2 monoclonal antiserum, 1∶1000 dilution; anti-*T. gondii* MIC4 rabbit antiserum, 1∶1000 dilution; anti-*T. gondii* VP1 rabbit antiserum, 1∶2000 dilution; anti-*T. gondii* BAG1 rabbit antiserum [68], 1∶250 dilution; anti-*T. gondii* P-gp minigene mouse antiserum, 1∶250 dilution. Fluorophore-conjugated secondary antibodies were used at 1∶200 dilution. Nuclei were visualized with 4′, 6-diamidino-2-phenylindole (DAPI).

Microscopy analyses were performed on a Leica DM IRBE fluorescence microscope or on a Leica SP2 AOBS confocal laser-scanning microscope (Leica Microsystems, Wetzlar, Germany), using the appropriate settings. Image stacks of optical sections were further processed using the Huygens deconvolution software package version 2.7 (Scientific Volume Imaging, Hilversum, NL).

### Transmission electron microscopy analysis

P-gp DKO host cells were grown in 75 cm^2^ flasks, infected with *T. gondii* tachyzoites at MOI 1 and incubated at 37°C for 24 h prior to fixation with 3% paraformaldehyde and 0.5% glutaraldehyde. Ultrathin sections were stained with anti-P-gp C219, 1∶2 dilution, followed by 10 nm gold conjugated anti-mouse antibodies, or double labeled with anti-P-gp C219, followed by 15 nm gold conjugated anti-mouse antibodies, and anti-VP1, 1∶250 dilution, followed by 5 nm gold conjugated anti-rabbit antibodies. Sections were counterstained with uranyl acetate and lead citrate and analyzed in a transmission electron microscope (CM12, Philips, Eindhoven, The Netherlands) equipped with a CCD camera (Ultrascan 1000; Gatan, Pleasanton, CA) at an acceleration voltage of 100 kV.

### Lipid analyses

For analysis of *T. gondii* lipid synthesis, purified parasites were labeled with 0.5 µCi/mL [^3^H]palmitic acid or 4 µCi/mL [^3^H]choline for 3 h in DMEM supplemented with 10% FCS. After extensive washing with PBS and 0.05% fat free BSA in PBS, lipids were extracted according to [69] and aliquots corresponding to equal protein content were separated by high performance thin layer chromatography (HPTLC) on Silica Gel 60 plates using chloroform/methanol/25% NH_4_OH (65∶25∶4.5). Radiolabeled bands were visualized by use of a tritium-sensitive screen (Perkin-Elmer, Boston, MA) in a Personal Molecular PhosphoImager FX (Biorad), identified according to co-migrating standards (Avanti Polar Lipids, Alabaster, Alabama) visualized by iodine vapors and quantified using ImageQuant software (Amersham, Otelfingen, Switzerland). For determination of radioactivity associated with total cell extract or lipid fraction, labeled cells aliquots were solubilized in 0.1N NaOH or lipid extracted as described above and radioactivity measured by liquid scintillation.

### Semi-quantitative real time-PCR (RT-PCR)

RNA was isolated from 10^7^ parasites isolated from WT or P-gp DKO host cells after five lysis cycles using an RNAeasy kit (Qiagen, Stanford, CA) following the “Animal Cells Spin” protocol. Residual genomic DNA was removed with DNase I digestion according to the manufacturer's protocol. First strand cDNA synthesis was performed using ∼250 ng RNA and Omniscript reverse transcriptase (Qiagen), according to manufacturer's protocol. Amplification was performed in an iCycler iQ (Biorad, Hercules, CA) Primer pairs (5′-3′ orientation) used for amplification of parasite tubulin (TUB), and P-gp were:

T.gTUB s CCAGGAGATGTTCAAG, T.gTUB as ACTCGGACACCAGGTCGTTC, T.gPgp s AAGGACAGCCGAAGGAAGAC, T.gPgp as GATGATGTCCGTCTGTGAC. Control PCR showed absence of contamination from host cell RNA or parasite genomic DNA. To assess the efficiency of the amplification reactions, standard curves for every primer pair and cDNA were generated from six-fold serial dilutions in duplicate, using the iQ5 software. Expression levels of the genes were given as values in arbitrary units relative to the amount of the constitutively expressed house-keeping gene tubulin.

### Western blot analysis

Cell lysates for immunoblots were prepared by sonicating cells at 10^7^/ml in 50 mM Tris-HCl (pH 6.8), 10% glycerol, 2% SDS, 5 mM DTT, 0.5 mM phenylmethylsulfonylfluoride and complete protease inhibitor mixture (Calbiochem). Samples corresponding to 40 µg proteins were mixed with SDS-PAGE loading buffer and incubated 10 min at room temperature to prevent P-gp aggregation. Samples were separated on 7.5% SDS-PAGE gels, transferred to nitrocellulose membranes, and probed using anti-P-gp C219 (1∶50), anti-*T. gondii* P-gp minigene (1∶1000) and anti-tubulin (1∶2000) antibodies. Immunoreactive bands were visualized with horseradish peroxidase-conjugated secondary antibodies and enhanced chemiluminescence (ECL).

For analysis of MIC2 secretion, 100 µL aliquots containing 7×10^7^ parasites were incubated with 10 µM GF120918 or 1 µM Ca^2+^ ionophore A23187 for 30 min at 37°C. 80 µL of supernatant were mixed with SDS-PAGE loading buffer, separated on 10%, SDS-PAGE gels, transferred to nitrocellulose membranes, and probed using anti-*T. gondii* MIC2 (1∶1000) and anti-tubulin (1∶2000) antibodies.

### Determination of protein concentration

Protein content was determined using the Bio-Rad Protein Assay according to the instructions provided by the manufacturer. Bovine serum albumin was used for the standard curve.

### Statistical Analyses

Data are expressed as means ± S.E. The statistical significance of differences in the means of experimental groups was determined using an unpaired, two-tailed Student's *t* test using GraphPad Prism 4.0c (GraphPad Software, Inc.) and a probability value <0.05 was considered statistically significant.

## Supporting Information

Figure S1 Detection of P-gp in T. gondii. A. Immunoblot analysis of T. gondii extracts probed with the P-gp specific monoclonal antibody C219 or with the anti-T. gondii P-gp minigene antibody. Tubulin staining was used as a loading control. Due to the low levels of P-gp expression compared with tubulin, probed membranes were exposed for different times to record unsaturated signal for both proteins. B. Protein sequence alignment of T. gondii P-gp (Tgmdr1) with the two mouse homologues. Highlighted in yellow are the sequences used to create the P-gp minigene fusion protein. Red box, sequence used in a previous study [1] to raise antibodies specific for T. gondii P-gp. Green box, epitope recognized by the P-gp specific monoclonal antibody C219. Gene bank accession numbers: T.g mdr1, DQ094188; Mmdr1a, NP_035206; Mmdr1b, NP_035205.
(2.96 MB TIF)

Figure S2 Detection of T. gondii MIC2 secretion. A. Immunoblot analysis of supernatant (SN) and pellets of T. gondii cultures incubated with 10 µM GF120918 (GF), 1 µM of Ca2+ ionophore A23187 (CaI) or solvent (cntl). Tubulin staining (Tub) was used as a loading control. Due to the low levels of basal MIC2 secretion compared with A23187- induced secretion, probed membranes were exposed for longer times (asterisk) to record unsaturated signal for both proteins. B. Densitometric quantification of the relative amount of secreted MIC2, normalized by the total protein amount, expressed as percentage of untreated control (cntl).
(0.90 MB TIF)
