# Supplementary figures and images for: The P-glycoprotein Inhibitor GF120918 Modulates Ca2+-Dependent Processes and Lipid Metabolism in Toxoplasma Gondii
Source: PLoS One. 2010 Apr 8;5(4):e10062. doi: 10.1371/journal.pone.0010062 (PMC2851653; doi:10.1371/journal.pone.0010062)

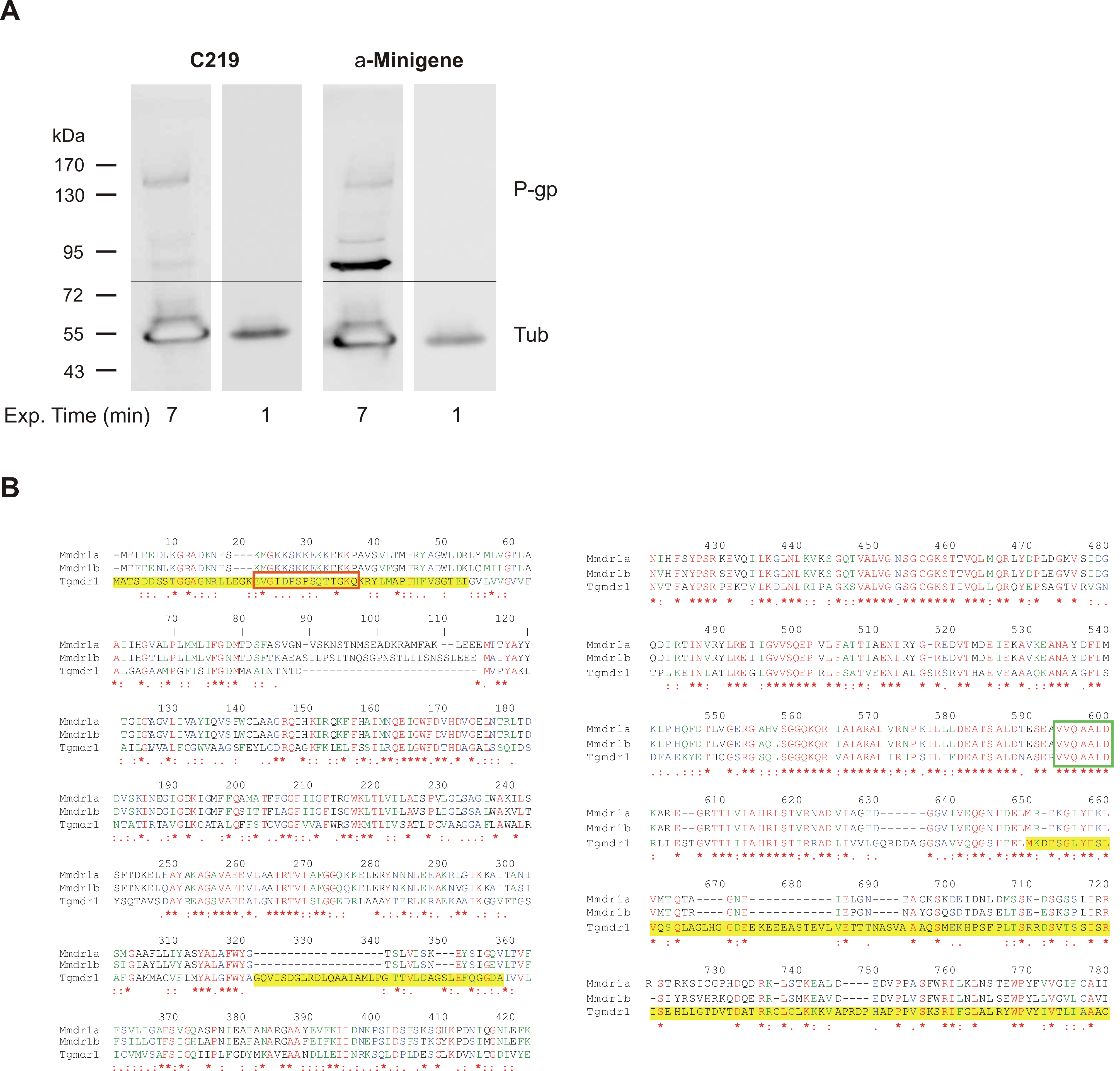

Supplement: Figure S1 — Detection of P-gp in T. gondii. A. Immunoblot analysis of T. gondii extracts probed with the P-gp specific monoclonal antibody C219 or with the anti-T. gondii P-gp minigene antibody. Tubulin staining was used as a loading control. Due to the low levels of P-gp expression compared with tubulin, probed membranes were exposed for different times to record unsaturated signal for both proteins. B. Protein sequence alignment of T. gondii P-gp (Tgmdr1) with the two mouse homologues. Highlighted in yellow are the sequences used to create the P-gp minigene fusion protein. Red box, sequence used in a previous study [1] to raise antibodies specific for T. gondii P-gp. Green box, epitope recognized by the P-gp specific monoclonal antibody C219. Gene bank accession numbers: T.g mdr1, DQ094188; Mmdr1a, NP_035206; Mmdr1b, NP_035205. (2.96 MB TIF) [file pone.0010062.s001.tif]

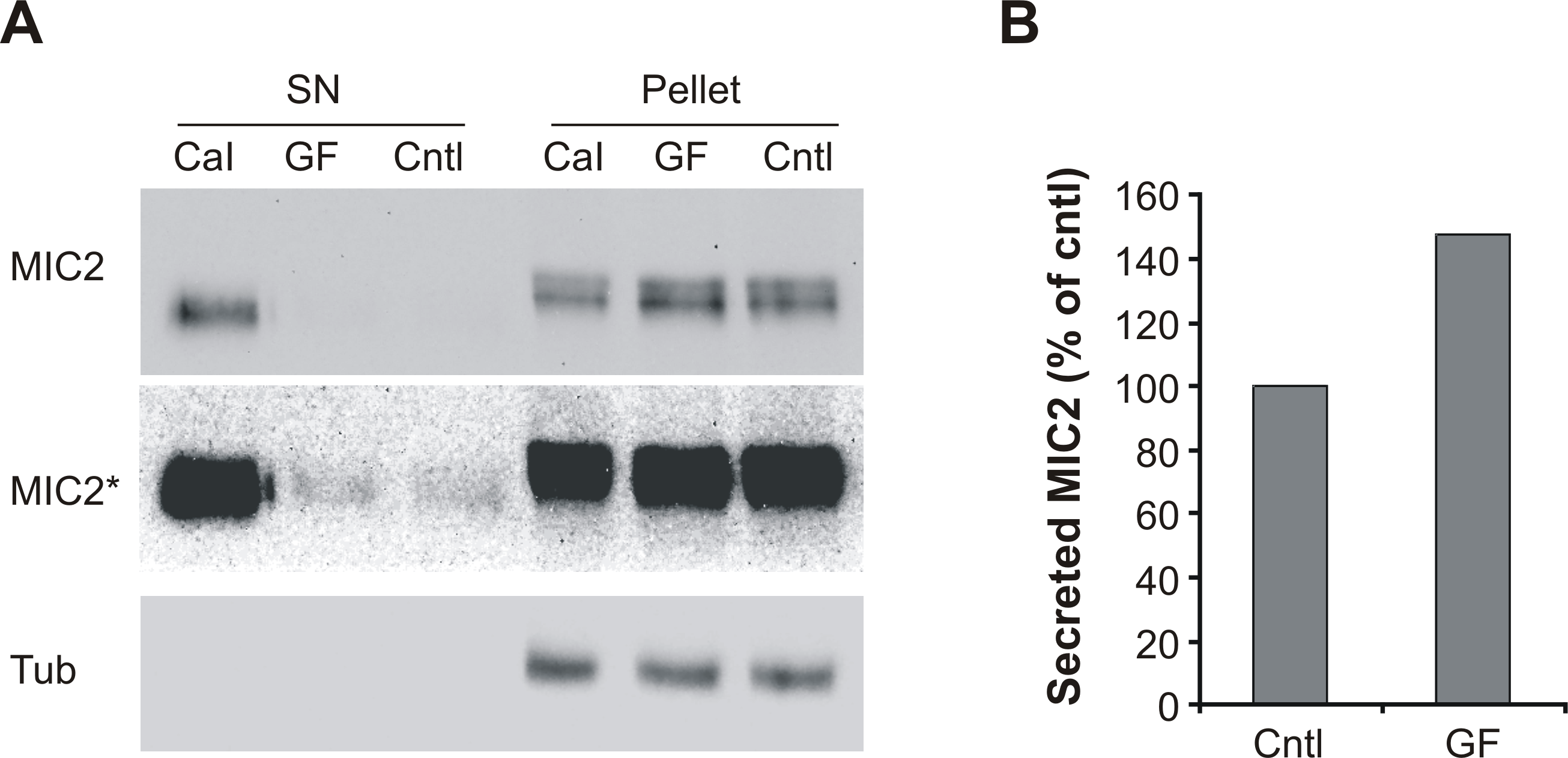

Supplement: Figure S2 — Detection of T. gondii MIC2 secretion. A. Immunoblot analysis of supernatant (SN) and pellets of T. gondii cultures incubated with 10 µM GF120918 (GF), 1 µM of Ca2+ ionophore A23187 (CaI) or solvent (cntl). Tubulin staining (Tub) was used as a loading control. Due to the low levels of basal MIC2 secretion compared with A23187- induced secretion, probed membranes were exposed for longer times (asterisk) to record unsaturated signal for both proteins. B. Densitometric quantification of the relative amount of secreted MIC2, normalized by the total protein amount, expressed as percentage of untreated control (cntl). (0.90 MB TIF) [file pone.0010062.s002.tif]
